# Supplementary material for: Operando investigation of the synergistic effect of electric field treatment and copper for bacteria inactivation
Source: Nat Commun. 2024 Feb 14;15:1345. doi: 10.1038/s41467-024-45587-3 (PMC10867087; doi:10.1038/s41467-024-45587-3)
Supplement: Supplementary file 1 — Supplementary Information [file 41467_2024_45587_MOESM1_ESM.pdf]

Supplementary information for

Operando Investigation of the Synergistic Effect of Electric Field Treatment and Copper for Bacteria Inactivation

Mourin Jarin[1], Ting Wang[1], Xing Xie[1,2*]

[1]School of Civil and Environmental Engineering, Georgia Institute of Technology, Atlanta, Georgia 30332, United States

[2]Institute for Electronics and Nanotechnology, Georgia Institute of Technology, Atlanta, Georgia, 30332, United States

[*]Corresponding author: Xing Xie, Email: xing.xie@ce.gatech.edu

Table of Contents

1. Supplementary Figures

Supplementary Figure 1. Overall inactivation percentage for Cu-only

Supplementary Figure 2. Inactivation percentage for pulse width 500 ns and Cu concentrations 0-2 mg/L

Supplementary Figure 3. Inactivation percentage for pulse width 1 μs and Cu concentrations 0-2 mg/L

Supplementary Figure 4. Inactivation percentage for pulse width 2 μs and Cu concentrations 0-2 mg/L

Supplementary Figure 5. Additional plots of inactivation percentage over 3 hours for all conditions

Supplementary Figure 6. The lethal electroporation threshold (LET) for EFT-Cu

Supplementary Figure 7. Schematic and terms of a pulsed electric field

2. Supplementary Note

1 Device operation without EFT

2 Fabrication and design of LOAC electrode curvature

3 LOAC precoating procedure

4 Parameter setup, procedure of pulse applications, and ROS observation and avoidance

5 Imaging and data processing through MATLAB

3. Supplementary References

1. Supplementary Figures

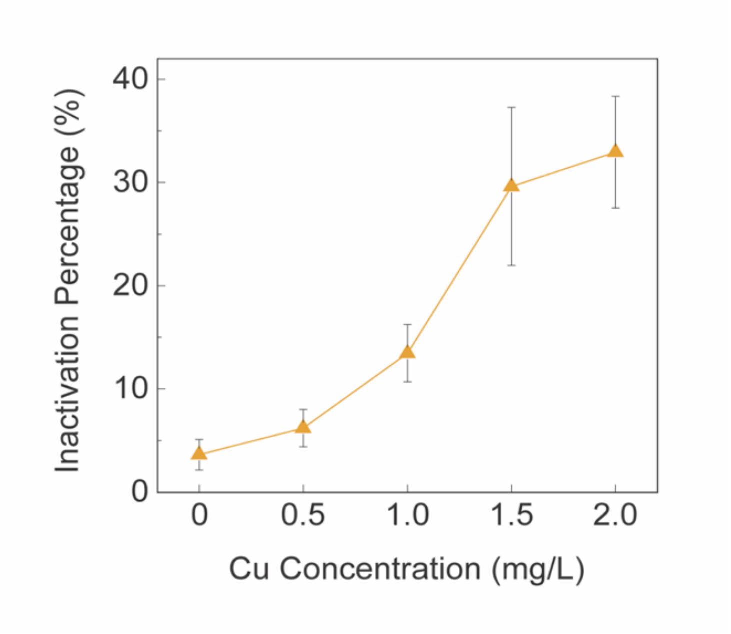

Supplementary Figure 1 | Overall inactivation percentage over increasing Cu concentration on the LOAC device without the application of EFT. Error bars represent standard deviation from the mean values of repeated experiments on 30 total replicate channels.

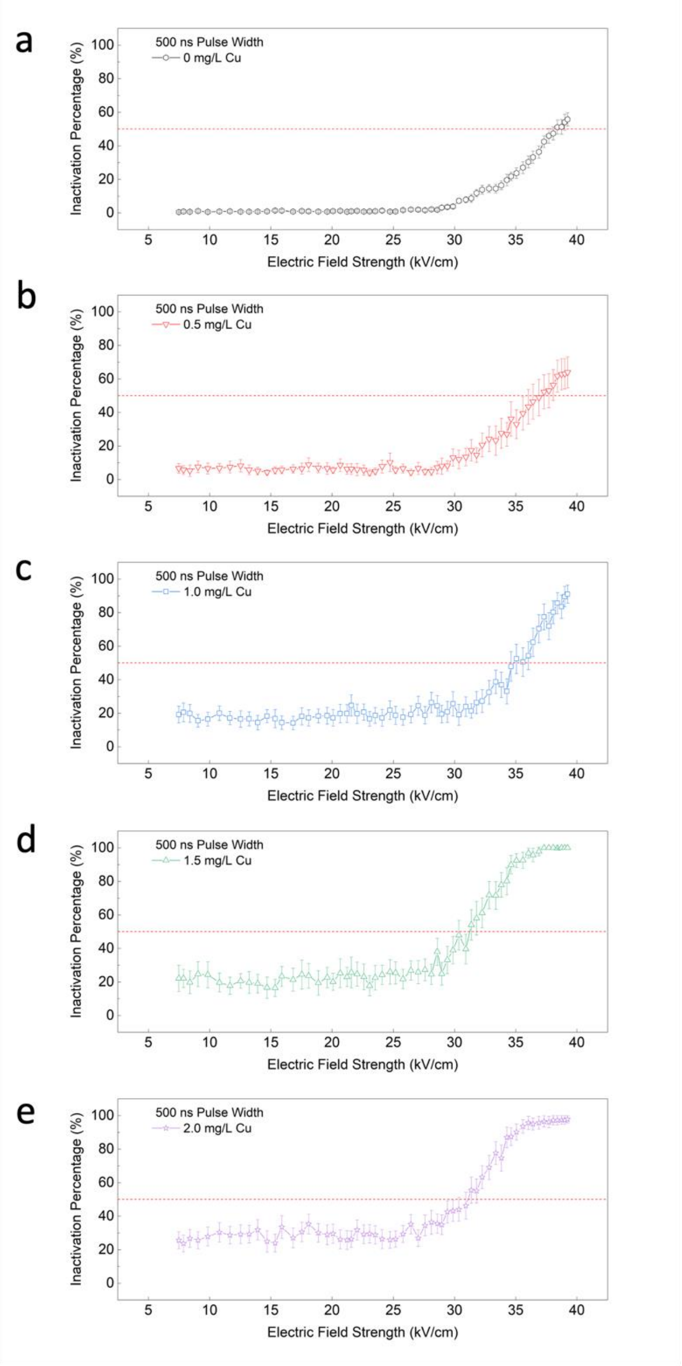

Supplementary Figure 2 | Inactivation percentage for pulse width 500 ns and Cu concentrations 0-2 mg/L. Error bars represent 95% confidence intervals from the mean values for all analyzed replicates out of a total 30 repeat channels for each individual experiment for Cu concentrations (**a**) 0 mg/L, (**b**) 0.5 mg/L, (**c**) 1.0 mg/L, **(d)** 1.5 mg/L, **(e)** 2.0 mg/L.

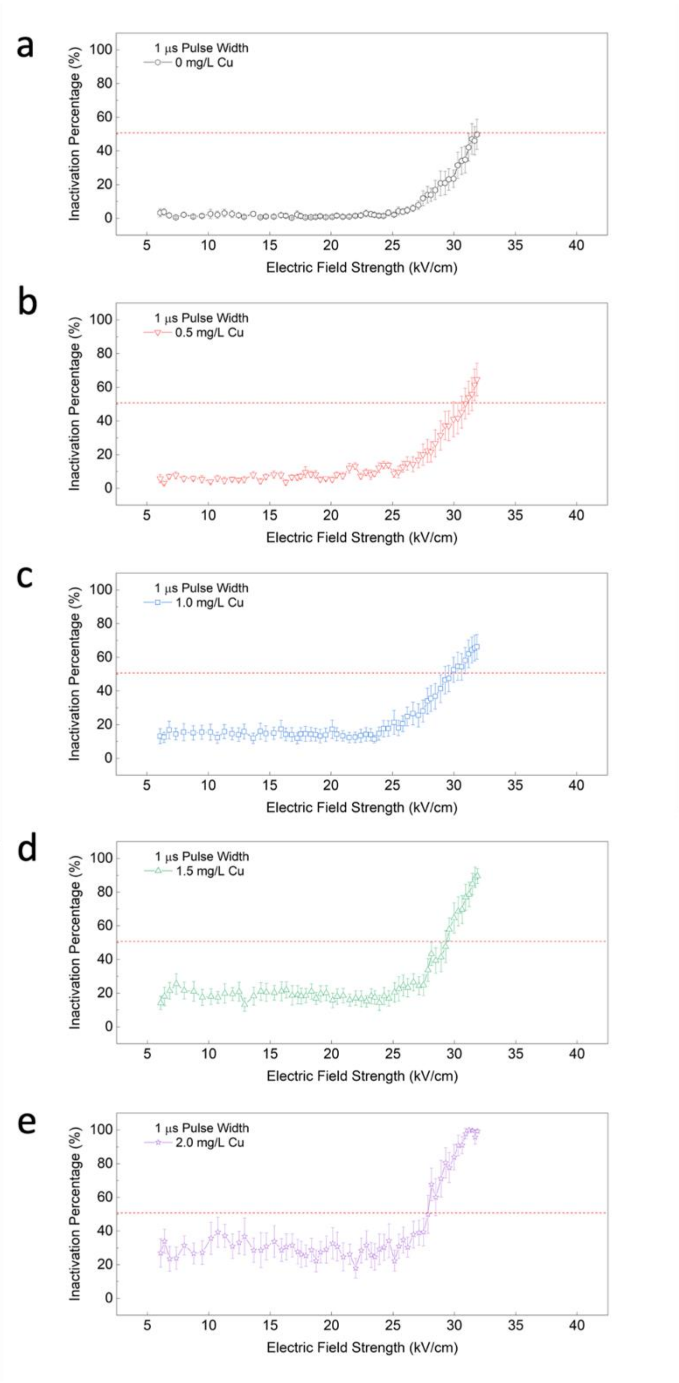

Supplementary Figure 3 | Inactivation percentage for pulse width 1 μs and Cu concentrations 0-2 mg/L. Error bars represent 95% confidence intervals from the mean values for all analyzed replicates out of a total 30 repeat channels for each individual experiment for Cu concentrations (**a**) 0 mg/L, (**b**) 0.5 mg/L, (**c**) 1.0 mg/L, **(d)** 1.5 mg/L, **(e)** 2.0 mg/L.

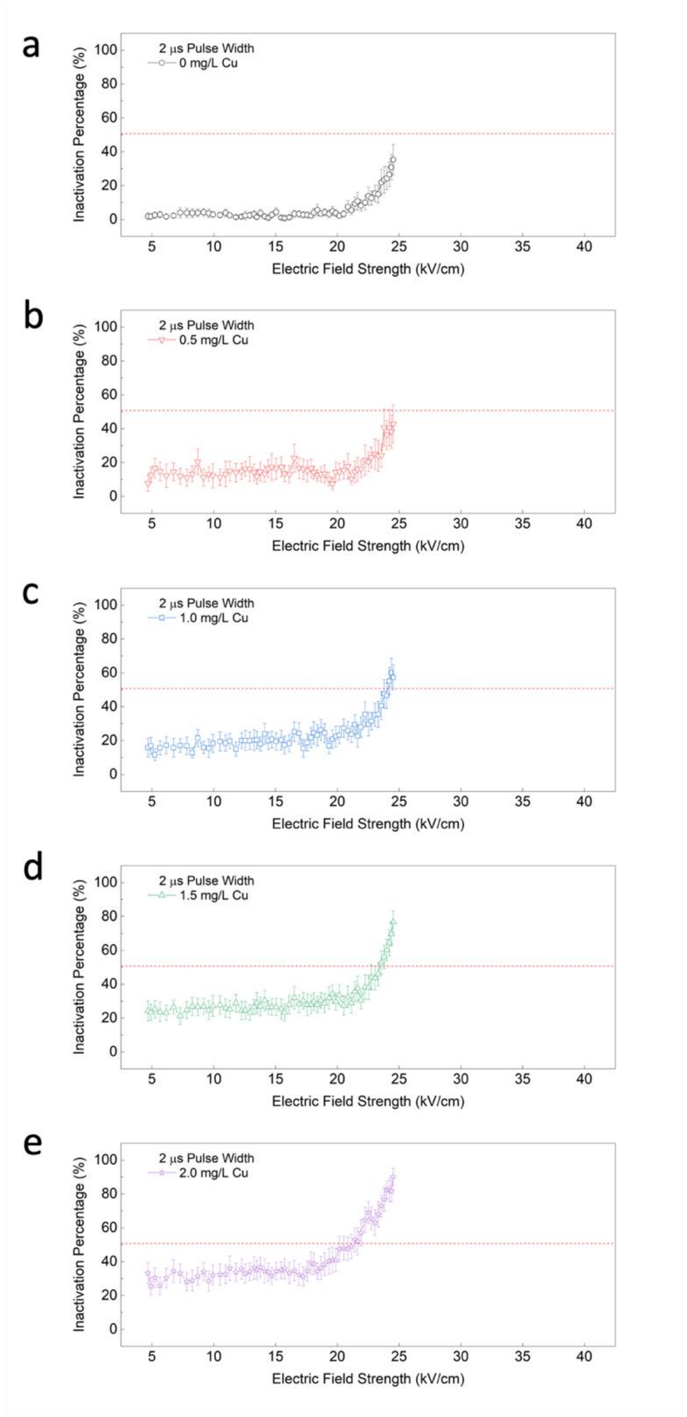

Supplementary Figure 4 | Inactivation percentage for pulse width 2 μs and Cu concentrations 0-2 mg/L. Error bars represent 95% confidence intervals from the mean values for all analyzed replicates out of a total 30 repeat channels for each individual experiment for Cu concentrations (**a**) 0 mg/L, (**b**) 0.5 mg/L, (**c**) 1.0 mg/L, **(d)** 1.5 mg/L, **(e)** 2.0 mg/L.

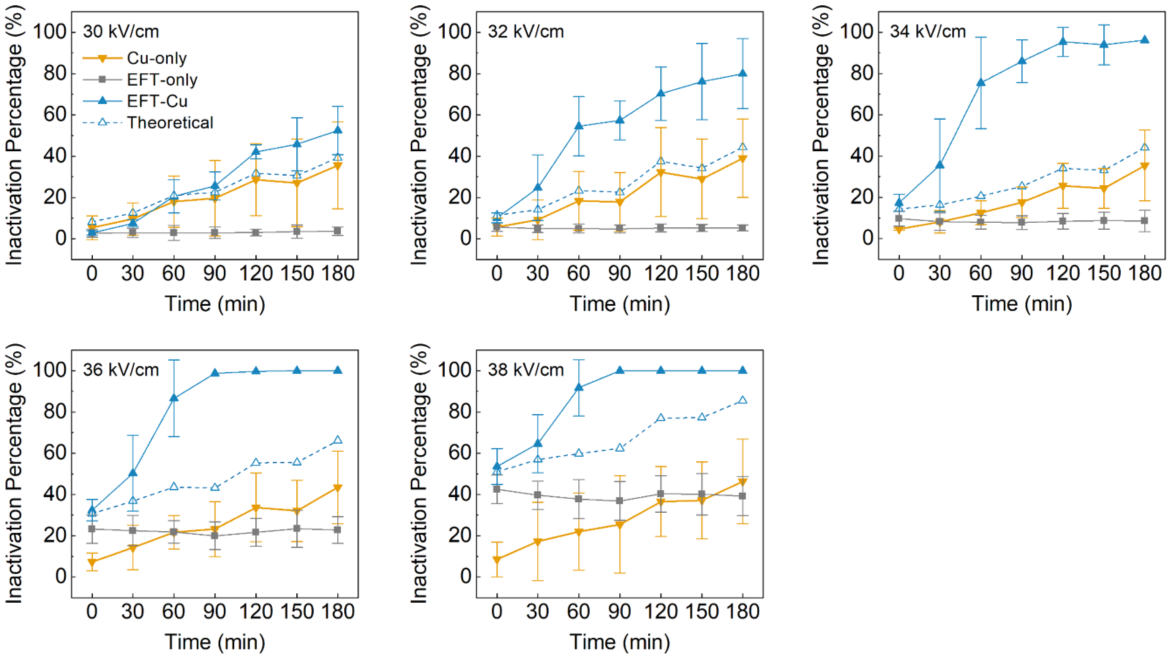

Supplementary Figure 5 | Additional and all plots for quantified inactivation percentage taken every 30 minutes over the course of 3 hours between 30-38 kV/cm electric field strength. The overall inactivation percentage over time in minutes for Cu-only, EFT-only, and EFT-Cu where the theoretical additive is calculated and compared to the measured EFT-Cu result. Individual plots are shown for specific electric field strengths, appearing in increasing order from top down and left to right. The error bars represent 95% confidence intervals from the mean values for the 5 total replicates collected and analyzed for each condition.

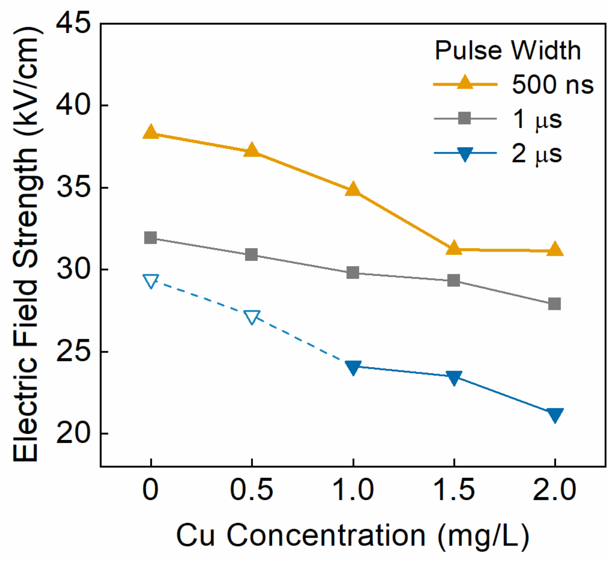

Supplementary Figure 6 | The lethal electroporation threshold (LET) for EFT-Cu. The electric field strength applied to reach the lethal threshold of 50% bacteria inactivation percentage (LET) is presented over the increasing Cu concentrations for three different pulse width conditions. For 2 μs pulse width (in blue) a portion is dashed here to represent the most likely values for LET, as the electric field strength necessary for 50% inactivation was likely within a range we consider ROS generation to also be involved, these values are resulted from experiments, but are not considered within the collective results of this study.

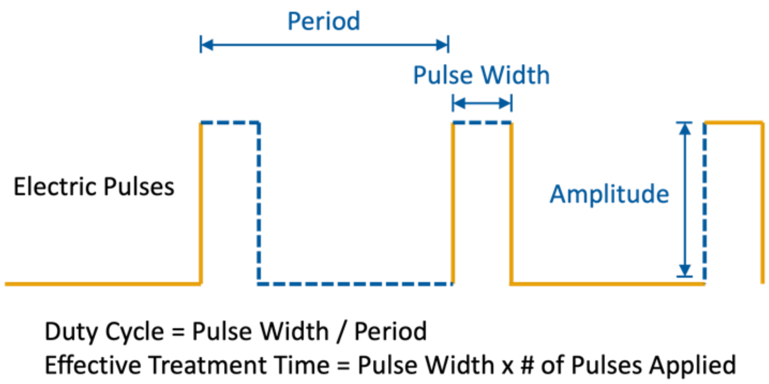

Figure S7 | Schematic and terms of a pulsed electric field.

2. Supplementary Note

Note 1 Device operation without EFT

The LOAC device was tested for its inactivation performance with exposure to Cu ion solution on the surface to determine how well the device could capture the death of model bacteria from Cu ion permeation alone. The inactivation percentage over concentration of Cu is shown in Fig. S1, where an increase is observed from 0-2 mg/L Cu. The results from our initial Cu tests show 1 mg/L Cu can induce ~15% of cell inactivation, while 2 mg/L Cu can have ~30%. These results suggest a clear and observable increase in inactivation on the chip surface without the EFT allowing us to confidently progress to observing a combination approach.

Note 2 Fabrication and design of LOAC electrode curvature

To summarize, the curvature is based off Eq. 1 (below) where x and w refer to half of the length and width of the channel, w_0 is half the width when $x = 0$, and k represents a constant.[1,2] The length of each channel was fabricated to be 440 µm and the narrowest portion that occurs at the center is 20 µm. Due to this intentional design, an applied electric field strength at any given point along the channel can be easily determined using Eq. 2 (below), where E is the electric field strength and U is the applied voltage. Because of this curved shape electrode edge (black portion), a linear strength electric field can be generated across the horizontal region with any applied voltage to the two bulk electrodes. The simulated linear electric field strength was demonstrated using COMSOL Multiphysics in a 3D model for an applied voltage of 80 V where the maximum strength is achieved ~40 kV/cm (**Fig. 1c** in the main text).

Equation 1: $w = w_0/(1-kx)$

Equation 2: $E = U/(2w) = U(1-kx)/2w_0$

Note 3 LOAC precoating procedure

Poly-L-lysine was used as the main ingredient for pre-coating the surface of the chip for bacteria immobilization. Poly-L-lysine was combined 1:1 with a 2 M pre-prepared borate buffer (composed of 3.1 g Boric acid (Millipore, cat# 100765) and 0.5 g NaOH (Millipore, cat# SX0593-1)) and dropped onto the center of the chip surface. In a closed dish to deter evaporation, the droplets were left for ~2 hours before being rinsed off gently with DI water, and then the chip was dried at 60 °C for 30 minutes.

Note 4 Parameter setup, procedure of pulse applications, and ROS observation and avoidance

For baseline Cu disinfection experiments, Cu solutions would be added to the chip surface and no EFT would be applied, but the same standard procedures of residual wait time and analysis were conducted. For conditions with both Cu and EFT involved, the Cu solutions were placed onto the chip surface during the application of pulses and remained on surface for the duration of residual wait time as well. For all experiments conducted in this study, it was important to avoid reactive oxygen species (ROS) and bubble generation, along with any significant heating effects during the application of electric pulses. Preliminary tests were completed to determine the conditions that observe ROS generation and strict boundaries were set on the electric fields for various pulse widths. To measure the oxidative stress, the cells were immobilized on the chip surface while being stained with a fluorescence dye DCFH-DA (Sigma-Aldrich, cat# D6883) at 0.2 mM concentration for 50 minutes with limited exposure to light.[3] Following successful immobilization, the excess dye and cell solution were gently rinsed off. When viewing under the fluorescence microscope, the cells will show a green fluorescence when there is oxidative stress while pulses are applied. The stronger the oxidative stress, the brighter the green fluorescence will show and in more cells. Several experiments were conducted using this setup for various operating parameters in order to

determine the threshold for ROS generation. Once determined and re-verified, all conditions for the results presented were set relatively below this threshold to ensure no oxidative stress was induced during our experimentation. In addition, all our experiments used operating parameters set to have a duty cycle of 1:1000. This means our rest time for any heat impacts to dissipate between pulses are 3 orders of magnitude longer than the individual pulse applied for all conditions. These parameters were determined through previous and preliminary studies to specifically limit and avoid factors like bubble formation and ROS generation, along with any significant heating as they can all impact the results. With these preventative measures in place, we are confident there is no concerns for heating on the device. All EFT-Cu experiments were performed to limit ROS generation, bubble formation, and heating so that we can be confident in the understanding of how Cu and EFT can work both independently and together without the interference of any other inactivation mechanisms potentially present.

Note 5 Imaging and data processing through MATLAB

Traditional PI staining was still used for all the experiments and results presented, but due to the varying operation needs for the time series observation, the dye staining method was slightly different for this part of the study. To serve the purpose of the time series observation, PI stain was added before the experimentation. The EFT occurred only within the first 20 seconds of the entire experiment, but some cells might be stained due to reversible electroporation. Since there was no shift observed in the EFT-only conditions after initial pulses were removed, we are confident if there was any reversible electroporation present it could only occur in the first 1 minute of the experiment. In addition to this, the EFT conditions shown in Figure 3 where stain was added before treatment (specific to the time stamp of 2 hours) resulted in similar and consistent data to those of the same conditions in Figure 2 where dye was added 2 hours later, only at the end of treatment.

This indicates further that the PI staining due to reversible electroporation is not significant and therefore not of high concern. Because of this, we consider the cells stained with PI as inactivated for the bulk of the results presented in Figure 3. Regarding the single cell study and results presented in Figure 4, the stain is also introduced before the experiment and pulse application. However, this part of the study is only focused on the individual cell, its cell membrane permeability, and initial rate of the observed fluorescence. Because of this, we are not concerned with the reversible pore closure or inactivation efficiency. We reference the concerns for reversible electroporation here to the reader for full context and understanding of the staining methods.

Regarding the MATLAB analysis briefly, the red rectangular portion in the middle of the channel, approximately 440 μm long and 20 μm high, was cropped in each image for every channel, and then vertically broken up into 120 columns, and the cell number in each column was counted using binary color coding in MATLAB. The inactivation efficiency in each column was then calculated by dividing the total number of inactivated cells (from the fluorescent image **Fig. 1f**) by the total number of cells present in each channel (from the DIC image **Fig. 1e**) in every column. As the channel is symmetrical, all quantified values have a duplicate. This analysis results in 60 values for each electric field strength data point shown in the results. The bulk of the results in the text reflect single data points that were calculated as the average of all 60 values gathered from each of 30 replicate channels. The data analysis was developed specifically for consistency and simplicity of results presented to the readers. For our single cell study specifically, both the Cu solution and propidium iodide (PI) were applied to the channels before EFT and took rapid images at frames for approximately every ~200 ms (fluorescent frames for EFT-only conditions due to the fast inactivation) and ~3 s (fluorescent and DIC frames for Cu-only and EFT-Cu conditions). These images and the intensity measurements of the fluorescence microscope were used to determine the

normalized fluorescence intensity. The cells with normalized values that reached 100% were considered completely saturated with the dye.

3. Supplementary References

1 Garcia, P. A., Ge, Z., Moran, J. L. & Buie, C. R. Microfluidic screening of electric fields for electroporation. *Scientific reports* **6**, 21238 (2016).

2 Weiss, N. G. *et al.* Dielectrophoretic mobility determination in DC insulator - based dielectrophoresis. *Electrophoresis* **32**, 2292-2297 (2011).

3 Yu, D. *et al.* Improved detection of reactive oxygen species by DCFH-DA: New insight into self-amplification of fluorescence signal by light irradiation. *Sensors and Actuators B: Chemical* **339**, 129878 (2021).
