# Supplementary material for: Operando investigation of the synergistic effect of electric field treatment and copper for bacteria inactivation
Source: Nat Commun. 2024 Feb 14;15:1345. doi: 10.1038/s41467-024-45587-3 (PMC10867087; doi:10.1038/s41467-024-45587-3)
Supplement: Supplementary file 3 — Description of Additional Supplementary Files [file 41467_2024_45587_MOESM3_ESM.pdf]

<u>Description of Additional Supplementary Files</u>

Supplemental Code 1

This file contains the MATLAB scripts used to analyze the data presented in this study.

Supplementary Movie 1 PI staining of cells treated with Cu-only

Video S1 | PI staining of cells treated with Cu-only. This snapshot from Video S1 using no EFT and only Cu ion dosage (2 mg/L) shows a zoomed in portion of the channel at the center observed for ~1 min. At the top displays the relative time for each channel image. PI_2 represents the time in seconds at which each fluorescent image was taken and DIC represents the time each differential interference contrast image was taken. Specific cells that are observed to be damaged and therefore permeable to PI-staining during this minute are circled in yellow.

Supplementary Movie 2 PI staining of cells treated with EFT-only

Video S2 | PI staining of cells treated with EFT-only. This snapshot from Video S2 uses EFT (2 ms pulse width, 2 ms period, 10k pulses, and 50 V) and Cu dosage (2 mg/L) to show a zoomed in portion of the channel at the center observed for ~1 min. The initial pulse application portion of both EFT-only and EFT-Cu are observed to be very similar, thus we only display one here. At the top displays the relative time for each channel image. PI_2 represents the time in seconds at which each fluorescent image was taken and DIC represents the time each differential interference contrast image was taken. Specific cells that are observed to be damaged and therefore permeable to PI-staining during the initial quick pulse application are circled in yellow.

Supplementary Video 3 PI staining of cells treated with EFT-Cu after pulses are removed

Video S3 | PI staining of cells treated with EFT-Cu after pulses are removed. This snapshot from Video S3 uses EFT (2 ms pulse width, 2 ms period, 10k pulses, and 50 V) and Cu dosage (2 mg/L) to show a zoomed in portion of the channel at the center a few minutes after pulses are removed. At the top displays the relative time for each channel image. PI_2 represents the time in seconds at which each fluorescent image was taken and DIC represents the time each differential interference contrast image was taken. Specific cells that are observed to be damaged and therefore

permeable to PI-staining post pulse application and by Cu ion permeation are circled in yellow.

Supplementary Movie 4 Single cell analysis through PI staining with Cu-only

Video S4 | Single cell analysis through PI staining with Cu-only. This snapshot from Video S4 shows the single cell staining of Cu-only condition over ~1 min. At the top displays the relative time for each channel image. PI_2 represents the time in seconds at which each fluorescent image was taken and DIC represents the time each differential interference contrast image was taken. The specific cell that is observed to be damaged and therefore permeable to PI-staining by Cu ion permeation is circled in yellow.

Supplementary Movie 5 Single cell analysis through PI staining with EFT-only

Video S5 | Single cell analysis through PI staining with EFT-only. This snapshot from Video S5 shows the single cell staining of EFT-only condition over a few seconds. At the top displays the relative time in seconds for each fluorescent image taken. The specific cell that is observed to be damaged and therefore permeable to PI-staining by rapid electroporation is circled in yellow.

Supplementary Movie 6 Single cell analysis through PI staining with EFT-Cu after pulses are removed.

Video S6 | Single cell analysis through PI staining with EFT-Cu after pulses are removed. This snapshot from Video S6 shows the single cell staining of EFT-Cu condition after pulses are removed. At the top displays the relative time for each channel image. PI_2 represents the time in seconds at which each fluorescent image was taken and DIC represents the time each differential interference contrast image was taken. The specific cell that is observed to be damaged and therefore permeable to PI-staining by Cu ion permeation; post pulse application is circled in yellow.
