# Supplementary material for: Operando investigation of the synergistic effect of electric field treatment and copper for bacteria inactivation
Source: Nat Commun. 2024 Feb 14;15:1345. doi: 10.1038/s41467-024-45587-3 (PMC10867087; doi:10.1038/s41467-024-45587-3)
Supplement: Supplementary file 10 — Supplementary Code 1 [file 41467_2024_45587_MOESM10_ESM.pdf]

Supplementary Code used for Data Analysis for

Operando Investigation of the Synergistic Effect of Electric Field Treatment and Copper for Bacteria Inactivation

Mourin Jarin[1], Ting Wang[1], Xing Xie[1,2*]

[1]School of Civil and Environmental Engineering, Georgia Institute of Technology, Atlanta, Georgia 30332, United States

[2]Institute for Electronics and Nanotechnology, Georgia Institute of Technology, Atlanta, Georgia, 30332, United States

[*]Corresponding author: Xing Xie, Email: xing.xie@ce.gatech.edu

MATLAB (version 2023a, MathWorks) Scripts for Microscopy Image Processing

Scripts for rotating and cropping image

```
clear;
close all;
clc
warning off

file_path_orig=([pwd,'/']);  %1 diff_images/']);
subdir=dir(file_path_orig);


final_folder = [file_path_orig, '/'];
mkdir(final_folder)
file_path_final = ([file_path_orig,'/']);

for iiii=3:length(subdir)

    %% ------------------ image rotation---------------------------

    folder=subdir(iiii).name;
    file_path =([file_path_orig,folder]);
    img_path_list=dir(strcat(file_path,'/','*.bmp'));
    img_num=length(img_path_list);

    folder_r=[folder,'_r'];
    new_folder=[file_path_orig,folder_r];
    mkdir(new_folder)

    for k=1:4:img_num-1
        image_name1 = img_path_list(k+1).name;
        image_name2 = img_path_list(k+2).name;
        image_name3 = img_path_list(k+3).name;
        image_name4 = img_path_list(k).name;
        I1=imread(strcat(file_path,'/', image_name1)); % Sytox Green image
        I2=imread(strcat(file_path,'/', image_name2)); % DIC image
        I3=imread(strcat(file_path,'/', image_name3)); % PI image
        I4=imread(strcat(file_path,'/', image_name4)); % stacked image


        %---- convert the DIC image from RGB to gray precision format -------

        GI2=rgb2gray(I2);
        [X,Y]=size(GI2); % Get the size of the image.[rows, columns]

        %---- find the middle area of the image-----

        Xi=floor(X/4);
        Xe=ceil(3*X/4);

        Yi=floor(Y/4);
        Ye=ceil(3*Y/4);
```

```matlab
        m=zeros(Ye-Yi+1,5);

        %-----find the edges of electrodes---------

        for j=Yi:Ye  % iterate through all columns
            for i=Xi:Xe   % iterate through all rows, find the electrode edge
                if GI2(i,j)  <=  30  &&  min(GI2(i+1:i+31,j))>30   % if row i is
black but the lower 10 rows are not black, row i is the edge

                    m(j-Yi+1,1)=j; % store the column number
                    m(j-Yi+1,2)=i; % store the row number of the top edge
                    break
                end
            end
        end
        for j=Yi:Ye
            for i=Xi:Xe
                if min(GI2(i-31:i-1,j))>30 && GI2(i,j)<=30 % if row i is black
but the top 10 rows are not black, row i is the edge
                    m(j-Yi+1,3)=i; % row number of the bottom edge
                    break
                end
            end
        end

        a=m(:,1); % a is the x coordinate
        b=m(:,2); % b is the y coordinate of the top electrode edge
        c=m(:,3); % c is the y coordinate of the bottom electrode edge

        % ------ fit the electrode edge to a quadratic function, then get the
y coordinate from the function -----

        f1 = polyfit(a,b,2);
        y1 = f1(1)*a.^2 + f1(2)*a +f1(3);

        f2 = polyfit(a,c,2);
        y2 = f2(1)*a.^2 + f2(2)*a +f2(3);


        % -------- find the angle of image rotation --------

        K(:,1)=diff(y1); % calculate the derivative at each point, i.e., the
difference between two adjacent points
        K(:,2)=diff(y2);
        K(:,3)=abs(K(:,1)-K(:,2));

        [~,position]=min(K(:,3));   % find the position where the two tangent
lines are parallel
        slop=K(position,1); % the slop is the derivative at the parallel place

        angle=slop*45;

        % g=polyfit(a,c,2);
```

```
        % h=polyval(g,a,1);

        %------- rotate the images -----------------

        R1 = imrotate(I1,angle,'bicubic','crop');
        R2 = imrotate(I2,angle,'bicubic','crop');
        R3 = imrotate(I3,angle,'bicubic','crop');
        R4 = imrotate(I4,angle,'bicubic','crop');

        imgname_1=sprintf( '%06d', k );
        imgname_2=sprintf( '%06d', k+1 );
        imgname_3=sprintf( '%06d', k+2 );
        imgname_4=sprintf( '%06d', k+3 );

        imwrite(R1,[new_folder,'/',imgname_1,'.bmp']);
        imwrite(R2,[new_folder,'/',imgname_2,'.bmp']);
        imwrite(R3,[new_folder,'/',imgname_3,'.bmp']);
        imwrite(R4,[new_folder,'/',imgname_4,'.bmp']);

    end

   %% ----- image crop------

    file_path = new_folder;
    img_path_list=dir(strcat(file_path,'/','*.bmp'));
    img_num=length(img_path_list);

    folder = folder_r;
    folder_f=[folder,'_f'];
    new_folder=[file_path_final,folder_f];
    mkdir(new_folder);
    img_index = 1;

    for k=1:4:img_num-1

        image_name1 = img_path_list(k+1).name;
        image_name2 = img_path_list(k).name;
        image_name3 = img_path_list(k+2).name;
        image_name4 = img_path_list(k+3).name;
        I1=imread(strcat(file_path,'/', image_name1)); % DIC     Sytox Green
image
        I2=imread(strcat(file_path,'/', image_name2)); % SYTOX  DIC image
        I3=imread(strcat(file_path,'/', image_name3)); % PI image
        I4=imread(strcat(file_path,'/', image_name4)); % stacked image

        GR1=rgb2gray(I1);
        [X,Y]=size(GR1); % Get the size of the image.[rows, columns]

        %---- find the middle area of the image-----

        Xi=floor(X/4);
        Xe=ceil(3*X/4);

        Yi=floor(Y/4);
```

```
        Ye=ceil(3*Y/4);

        edge = [];

        % ------ find the edge of new image ------

        for j=Yi:Ye
            for i=Xi:Xe
                if GR1(i,j)<=10 && min(GR1(i+1:i+11,j))>10
                    edge = [edge, i]; % store the row number in matrix m
                    break
                end
            end
        end


        x0=max(edge)+10; % find the lowest point of the top electrode edge and
slightly adjust downward by 10 pixels

        % ---- crop the channel part from the image ----

        rect=[1,x0,Y,70]; % a rectangle: [first x coordinate (column number,
first y coordinate (row number), width, height)
        C1=imcrop(I1,rect);
        C2=imcrop(I2,rect);
        C3=imcrop(I3,rect);

        %% ------ plot and save the new images -------------
        % ---- plot original images and channels -----

        %figure();
        %subplot(221);imshow(I1);
        %axis on;
        %rectangle('Position',rect,'LineWidth', 1,'EdgeColor','r')
        %subplot(222), imshow(I2);
        %rectangle('Position',rect,'LineWidth', 1,'EdgeColor','r')

        %subplot(223), imshow(C1);
        %subplot(224), imshow(C2);


        % -- save channels as new images for cell counting -----

        imgname_1=sprintf('%03d', img_index );
        imgname_2=sprintf('%03d', img_index+1 );
        imgname_3=sprintf('%03d', img_index+2 );

        imwrite(C1,[new_folder,'/',imgname_1,'.bmp']);
        imwrite(C2,[new_folder,'/',imgname_2,'.bmp']);
        imwrite(C3,[new_folder,'/',imgname_3,'.bmp']);

        img_index = img_index+3;
    end
end
```

Scripts for calculating inactivation efficiency

```
close all
clear;
clc;
warning off;

threshold = 50;
grid_num = 120;   % divide each image into 120 grids
img_width = 440;  % image is 440 um wide
um_per_grid = 440/grid_num; % width per grid in um
results={};

Line = importdata('/');  % import electric field data

file_path_orig=([pwd,'/']);
subdir=dir(file_path_orig);

for iiii=3:length(subdir)

    folder=subdir(iiii).name
    results{iiii-2,1}=folder;
    file_path =([file_path_orig,folder]);
    img_path_list=dir(strcat(file_path,'/','*.bmp'));
    img_num=length(img_path_list);

    %% --------- cell count in each image --------

    cell_count = zeros(img_num,grid_num);
    area_matrix = zeros(1,img_num*grid_num);  % area of each cell

for k = 1:img_num

        image_name = img_path_list(k).name;
        Ii=imread(strcat(file_path,'/', image_name));

        if rem(k,3) == 2
            R=Ii(:,:,2);
        else
            R=Ii(:,:,1);
        end

        %-----Otsu's method -------------

        R_double=im2double(R);  % Convert image to double precision

        if rem(k,3) == 1
            level=graythresh(R_double);  % threshold for DIC image
        else
            level=graythresh(R_double)+0.1;  % threshold for SYTOX and PI image
        end
```

```
        R_bw=imbinarize(R_double,level); % binarize to either 0 or 1
        I=R_bw;

        %figure()
        %subplot(211), imshow(Ii) % show the initial and binarized images
        %subplot(212), imshow(I)

        %----use watershed to separate cells ------------

        D=-bwdist(~I);
        mask=imextendedmin(D,0.1);

        D2=imimposemin(D,mask);

        Ld=watershed(D2);
        Water_splited=I;
        Water_splited(Ld==0)=0;
        I=Water_splited;

        %----set grid and count number in each column------

        [x,y]=size(I);
        pixel_per_grid=floor(y/grid_num);      % number of pixels per grid

        %global N;
        for q=1:grid_num % iterate each grid
            Iq=I(:,((q-1)*pixel_per_grid+1):(pixel_per_grid*q),:);     %  each
grid

            [areas_PI,L,N]=bwboundaries(Iq,'noholes');

            cell_count(k,q)=N;  % store the cell number in grid q image k

            %-----record area for each cell in all grids from all images-----

            for i =1:N
                item_area=size(areas_PI{i},1);
                area_matrix(i,((k-1)*grid_num+q))=item_area;   %  row:  cells;
column: all grids from all images
            end
        end
    end


%% --------- adjust cell count in each image and get percentage-------

    cell_count_new = cell_count;
    percent= [];

    for ii=1:3:img_num-1 % the DIC image

        for i = ii:ii+2 % DIC, sytox green, PI
```

```
%-----find the average cell area-----

            areas=nonzeros(area_matrix(:,(i-1)*grid_num+1:i*grid_num));
            table=tabulate(areas);
            if ~isempty(table)
            [F1,mostFreq_areas]=max(table(:,2));   % ERROR when table is empty
set, skip and make = 10
            mostFreq_area=mean(mostFreq_areas(:,1));     %this is if there are
more than one max number
            else mostFreq_area = 10;
            end

           %---- adjust cell count by cell area--------

            for x=(i-1)*grid_num+1:i*grid_num    % columns in area_matrix (each
grid in DIC images)
                j=x-(i-1)*grid_num;
                for y=1:length(area_matrix(:,x))  % rows (each cell)
                    if area_matrix(y,x)>mostFreq_area
                        %If the cell area is much larger than the average cell
area

                        n = area_matrix(y,x)/mostFreq_area;
                        % The large cell is n times larger than the average
cell area
                        cell_count_new(i,j)=cell_count_new(i,j)+round(n)-1;  %
row: image number, column: grid number
                        % Add n-1 cells
                    end
                end
            end


          % ----- calculate percentage -------------

            if i ~= ii
                percent                                                 =[percent;
cell_count_new(i,:)./cell_count_new(ii,:)*100];
            end
        end
    end

    percent(percent(:,:)>100) = 100;
    percent(isinf(percent)) = 0;

    row_count = size(percent,1);


  %% Analyze for Inactivation %%

    PI_percent = percent(2:2:end,:);    %matrix of just PI inactivation
    PI_mean = mean(PI_percent);         %mean of each section on grid
    PI_mean1 = transpose(PI_mean);
```

```
    EF_data = Line(:,2);             %Just the EF data
    n2 = height(EF_data)/grid_num; % average every n value all EF data over the
grid blocks
    Avg_EF = EF_data(n2/2:n2:end,:);    %the midpoint EF value for each section
of grid
    Distance = Line(:,1);
    Dis = Distance(n2/2:n2:end,:);      %chip distance midpoints
    half_Dis = Dis(1:1:grid_num/2,:);

    PI_2 = [PI_mean1(1:1:grid_num/2,:),flip(PI_mean1(grid_num/2+1:1:end,:))];
    PI_avg = mean(PI_2,2);      %60X1 of averaged values
    half_EF = Avg_EF(1:1:grid_num/2,:);

    Results = [Dis(:,1),Avg_EF(:,1),(PI_mean1(:,1))];    %matrix of um distance
on chip, EF value, and mean inactivation
    display(PI_mean1(:,1))
    %calculated over the grid number
    Results_Avg = [half_Dis(:,1),half_EF(:,1),(PI_avg(:,1))];
    display(PI_avg(:,1))

end
```
