# Supplementary material for: Operando investigation of the synergistic effect of electric field treatment and copper for bacteria inactivation
Source: Nat Commun. 2024 Feb 14;15:1345. doi: 10.1038/s41467-024-45587-3 (PMC10867087; doi:10.1038/s41467-024-45587-3)
Supplement: Supplementary file 11 — Reporting Summary [file 41467_2024_45587_MOESM11_ESM.pdf]

nature portfolio

Corresponding author(s): Xing Xie

Last updated by author(s): Jan 5, 2024

Reporting Summary

Nature Portfolio wishes to improve the reproducibility of the work that we publish. This form provides structure for consistency and transparency in reporting. For further information on Nature Portfolio policies, see our Editorial Policies and the Editorial Policy Checklist.

Statistics

For all statistical analyses, confirm that the following items are present in the figure legend, table legend, main text, or Methods section.

n/a	Confirmed	
☐	☒	The exact sample size (*n*) for each experimental group/condition, given as a discrete number and unit of measurement
☐	☒	A statement on whether measurements were taken from distinct samples or whether the same sample was measured repeatedly
☒	☐	The statistical test(s) used AND whether they are one- or two-sided<br>*Only common tests should be described solely by name; describe more complex techniques in the Methods section.*
☐	☒	A description of all covariates tested
☐	☒	A description of any assumptions or corrections, such as tests of normality and adjustment for multiple comparisons
☐	☒	A full description of the statistical parameters including central tendency (e.g. means) or other basic estimates (e.g. regression coefficient) AND variation (e.g. standard deviation) or associated estimates of uncertainty (e.g. confidence intervals)
☒	☐	For null hypothesis testing, the test statistic (e.g. *F*, *t*, *r*) with confidence intervals, effect sizes, degrees of freedom and *P* value noted<br>*Give P values as exact values whenever suitable.*
☒	☐	For Bayesian analysis, information on the choice of priors and Markov chain Monte Carlo settings
☒	☐	For hierarchical and complex designs, identification of the appropriate level for tests and full reporting of outcomes
☒	☐	Estimates of effect sizes (e.g. Cohen's *d*, Pearson's *r*), indicating how they were calculated

Our web collection on statistics for biologists contains articles on many of the points above.

Software and code

Policy information about availability of computer code

Data collection	No software was used for data collection.
Data analysis	A MATLAB code was developed using version 2023a, Mathworks to analyze the results. Our data was collected as fluorescence microscope images and analyzed using image analysis through MATLAB. These scripts are available with the paper.

For manuscripts utilizing custom algorithms or software that are central to the research but not yet described in published literature, software must be made available to editors and reviewers. We strongly encourage code deposition in a community repository (e.g. GitHub). See the Nature Portfolio guidelines for submitting code & software for further information.

Data

Policy information about availability of data

All manuscripts must include a data availability statement. This statement should provide the following information, where applicable:

- Accession codes, unique identifiers, or web links for publicly available datasets
- A description of any restrictions on data availability
- For clinical datasets or third party data, please ensure that the statement adheres to our policy

All data supporting the findings of this study are available within the article and its supplementary files. Any additional requests for information can be directed to, and will be fulfilled by, the corresponding authors. Source data are provided with this paper.

Research involving human participants, their data, or biological material

Policy information about studies with human participants or human data. See also policy information about sex, gender (identity/presentation), and sexual orientation and race, ethnicity and racism.

Reporting on sex and gender	NA
Reporting on race, ethnicity, or other socially relevant groupings	NA
Population characteristics	NA
Recruitment	NA
Ethics oversight	NA

Note that full information on the approval of the study protocol must also be provided in the manuscript.

Field-specific reporting

Please select the one below that is the best fit for your research. If you are not sure, read the appropriate sections before making your selection.

☒ Life sciences ☐ Behavioural & social sciences ☐ Ecological, evolutionary & environmental sciences

For a reference copy of the document with all sections, see nature.com/documents/nr-reporting-summary-flat.pdf

Life sciences study design

All studies must disclose on these points even when the disclosure is negative.

Sample size	A lab-on-a-chip device is applied in this study to conduct most of the experiments. On each chip, there are 30 replicate channels. The device was designed to optimize as much space for repeat experiments through these replicate channels without being too large to limit other operating parameters. According to the statistical analysis shown in the manuscript, the data collected are enough to support our findings in this study. Error bars indicating the 95% confidence intervals have been shown in the figures when appropriate.
Data exclusions	For the 30 channels on one chip, occasionally, some channels (5-10) are excluded in the analysis due to inconsistent connection to the circuit or if any obvious damage is present on the channel. This can occur through natural wear and tear of the device in experimentation. In any of these cases, those specific channels are not analyzed and also accounted for in the total number of replicates for any statistical analysis accordingly.
Replication	For each individual condition tested (for example under 1 voltage and 1 concentration), there were 30 total replicate channels to provide repeat results. The various operating parameters we tested for the bulk of the data presented were for 3 separate voltages and 5 different concentrations resulting a total of 15 different individual operating conditions. Each had a total of 30 replicate channels. According to the resulting trends and statistical analysis shown in the manuscript, the data replication and reproducibility are successful.
Randomization	This study aims to inactivate bacteria using a pulsed electric field and copper ions, therefore randomization is not relevant to our study. Additionally, the operating parameters and experimental conditions were determined through preliminary studies to limit any other factors that can contribute to the results unintentionally such as reactive oxygen species generation, bubble formation, and heating. The set variables in the study were chosen to mitigate these other possible mechanism and a range of voltages, pulse widths, and copper concentrations were chosen based on those preliminary results.
Blinding	This study aims to inactivate bacteria using a pulsed electric field and copper ions, therefore blinding is not relevant to our study. Additionally, we set strict and consistent procedures for all experiments using the lab-on-a-chip device and fluorescence microscope. The MATLAB code used for analysis was also specifically formulated to run the same for all experiments and images collected needing minimal human inputs once the results are collected.

Reporting for specific materials, systems and methods

We require information from authors about some types of materials, experimental systems and methods used in many studies. Here, indicate whether each material, system or method listed is relevant to your study. If you are not sure if a list item applies to your research, read the appropriate section before selecting a response.

Materials & experimental systems

n/a	Involved in the study
☒	☐ Antibodies
☒	☐ Eukaryotic cell lines
☒	☐ Palaeontology and archaeology
☒	☐ Animals and other organisms
☒	☐ Clinical data
☒	☐ Dual use research of concern
☒	☐ Plants

Methods

n/a	Involved in the study
☒	☐ ChIP-seq
☒	☐ Flow cytometry
☒	☐ MRI-based neuroimaging

Plants

Seed stocks	NA
Novel plant genotypes	NA
Authentication	NA
